# Supplementary figures and images for: Host casein kinase 1-mediated phosphorylation modulates phase separation of a rhabdovirus phosphoprotein and virus infection
Source: eLife. 2022 Feb 22;11:e74884. doi: 10.7554/eLife.74884 (PMC8887900; doi:10.7554/eLife.74884)

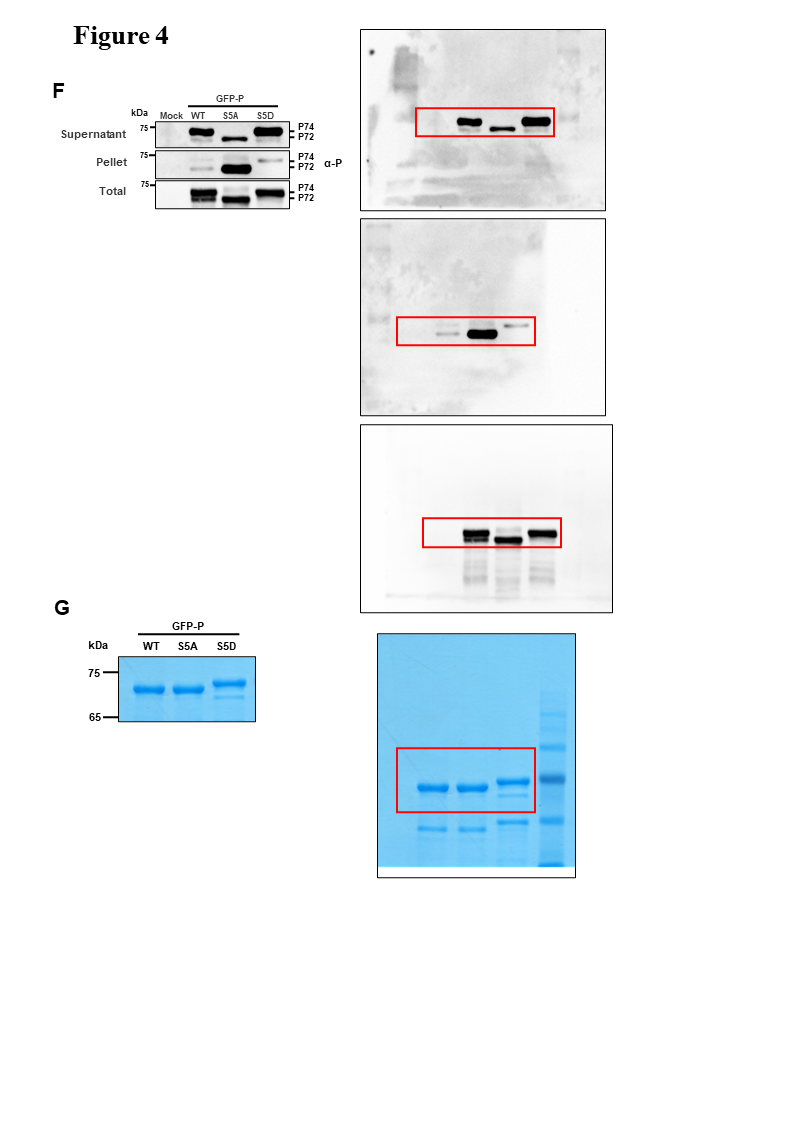

Supplement: Figure 4—source data 1. [file elife-74884-fig4-data1.zip › Figure 4-source datas/Figure 4-source datas 3.tif]

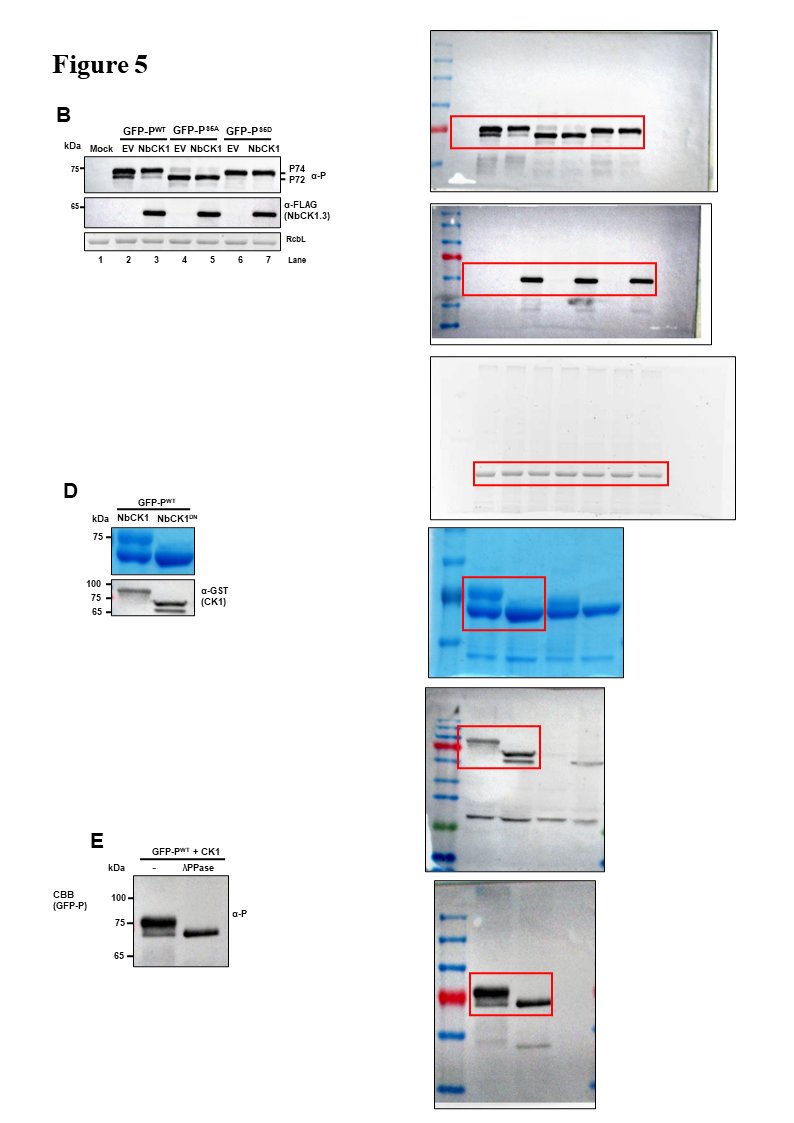

Supplement: Figure 5—source data 1. [file elife-74884-fig5-data1.zip › Figure 5-source datas/Figure 5-source datas.tif]

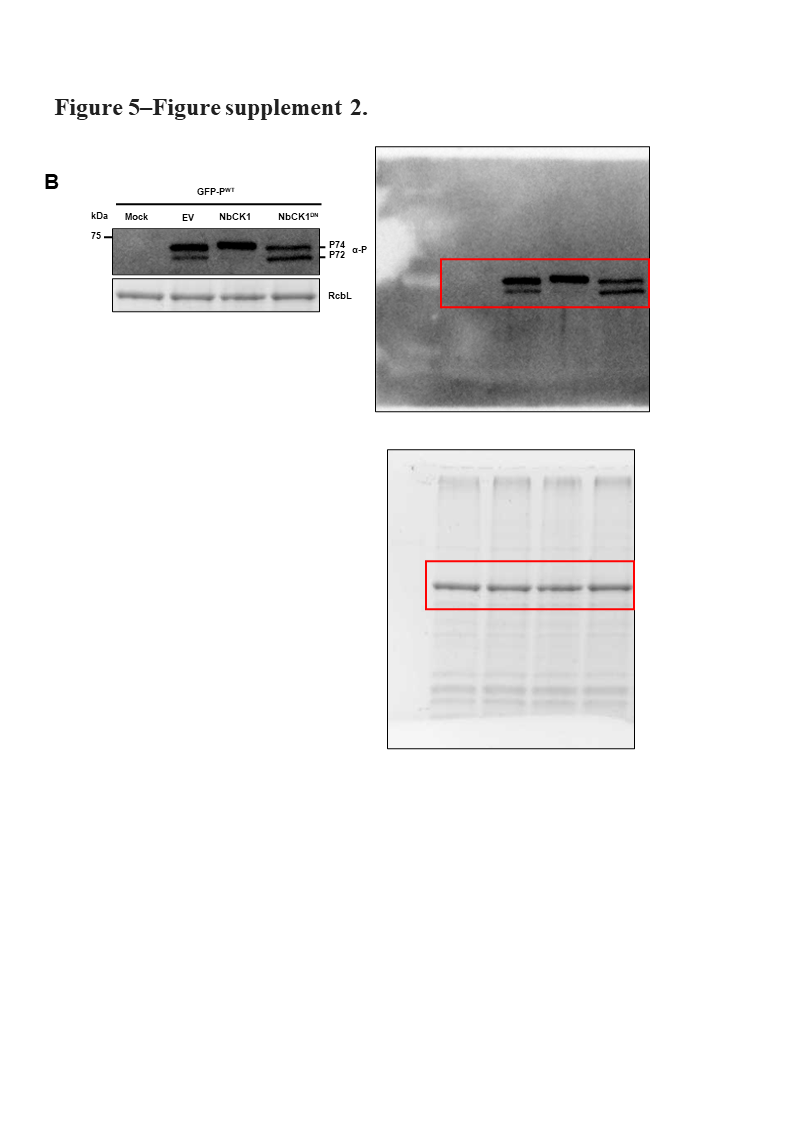

Supplement: Figure 5—figure supplement 2—source data 1. [file elife-74884-fig5-figsupp2-data1.tif]

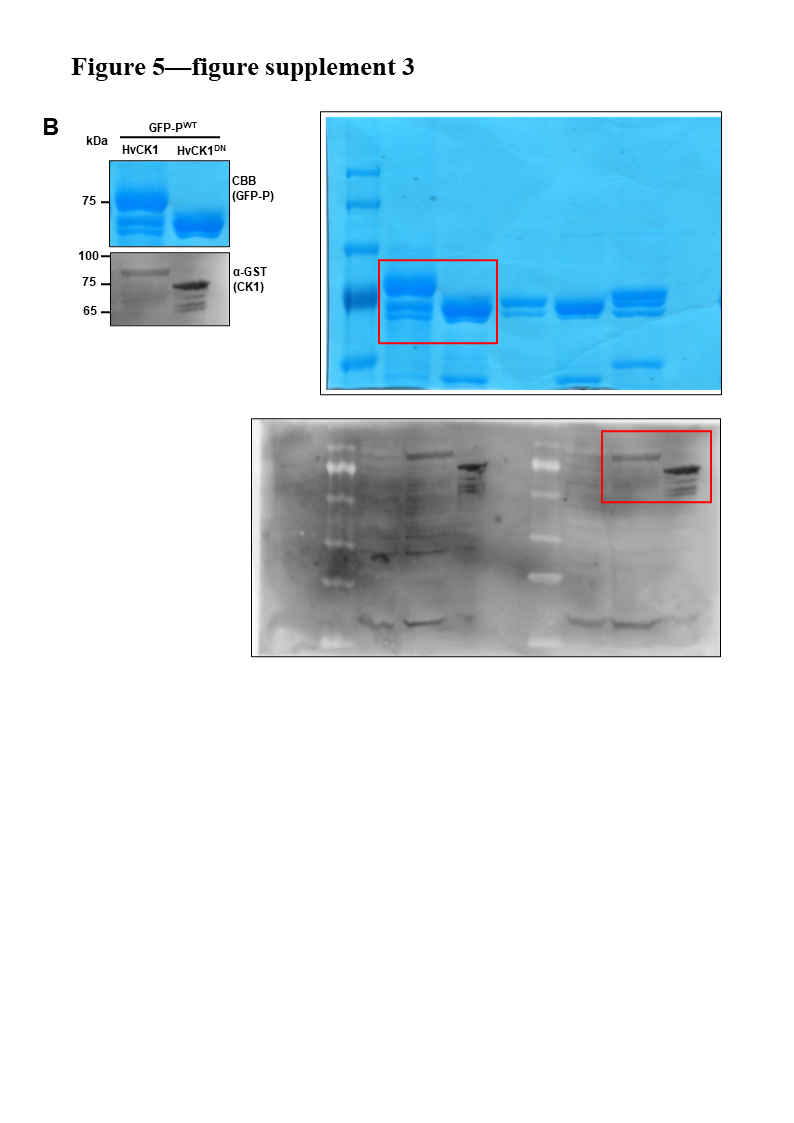

Supplement: Figure 5—figure supplement 3—source data 1. [file elife-74884-fig5-figsupp3-data1.tif]

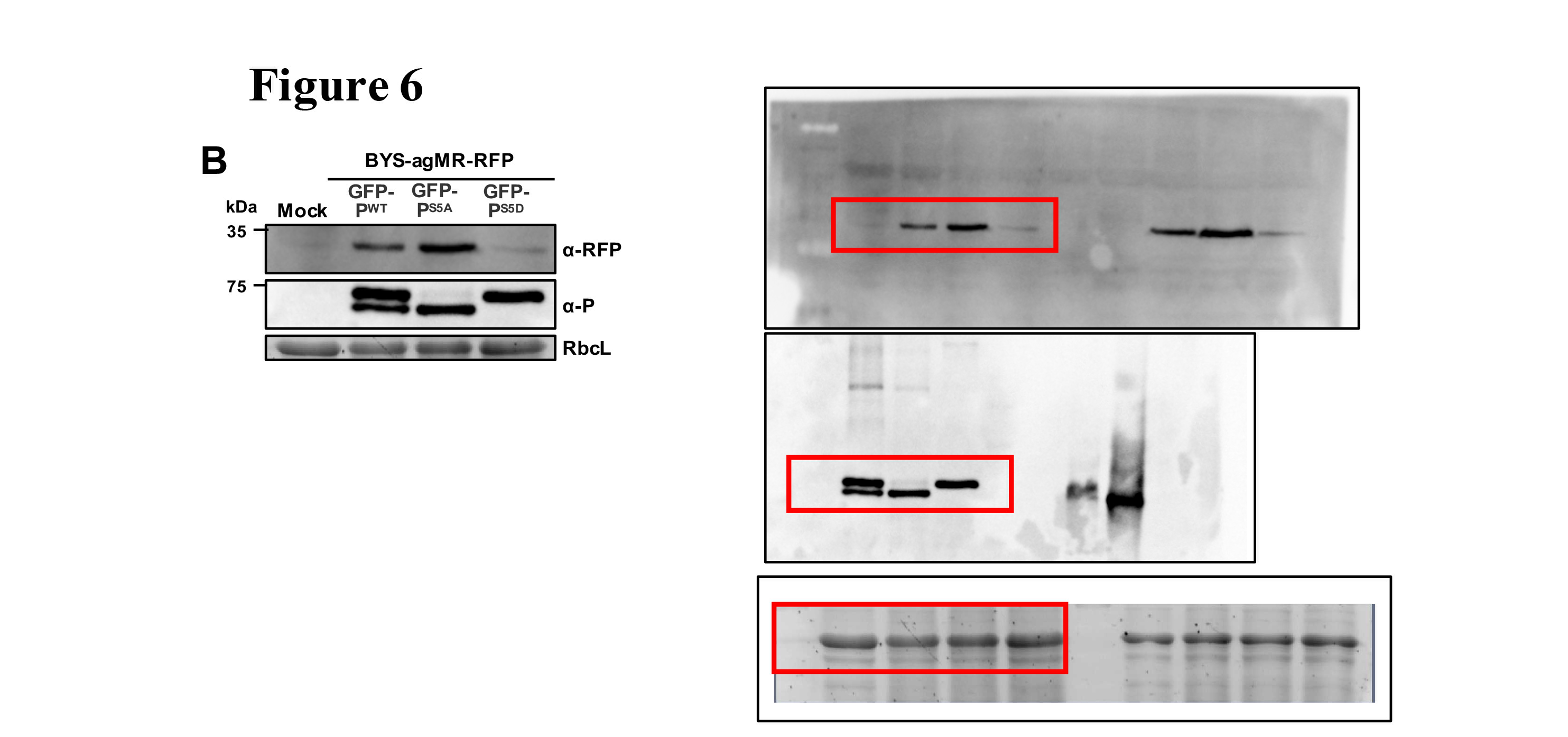

Supplement: Figure 6—source data 1. [file elife-74884-fig6-data1.zip › Figure 6-source datas/figure 6-source datas.tif]
