# Supplementary material for: Host casein kinase 1-mediated phosphorylation modulates phase separation of a rhabdovirus phosphoprotein and virus infection
Source: eLife. 2022 Feb 22;11:e74884. doi: 10.7554/eLife.74884 (PMC8887900; doi:10.7554/eLife.74884)
Supplement: Supplementary file 1. [file elife-74884-supp1.docx]

**Supplementary File 1. Primers used in this study**

| **Number** | **Primer** | **Primer sequence** | **Restriction Enzyme** | **Purpose** |
| --- | --- | --- | --- | --- |
| 1 | BYS agMR RFP F | AAGTAAAGGAGAAGAACTTTTCACTGGAG | inverse PCR | pBYSMV-agMR-RFP |
| 2 | BYS agMR RFP R | CATCTTTCTGGAAACGTCAAAAATAGAAACAG |  |  |
| 3 | BYS P-PGDG F | CTCGAGCTCAAGCTTCGAATTCTGCAGTCGACATGAGCTCATCCAATGCTGCAGTC | *Sal*I | pGDG-P^WT^, pGDG-P^S5A^, pGDG-P^S5D^ |
| 4 | BYS P-PGDG R | CGGTGGATCCCGGGCCCGCGGTACCGTCGACTTAGAGATCTCCATAGGGATCATGACGGG |  |  |
| 5 | spGFP11 F | CTACCGGTCGCCACCATGCGTGACCACATGGTCCTTCATG | inverse PCR | pGD-spGFP1-10-P |
| 6 | spGFP11 R | GAGATCTGAGTCCGGATTTGCCGCCAGTTATACCCGCC |  |  |
| 7 | PGDG F | GTATAACTGGCGGCAAATCCGGACTCAGATCTCGAGCTCAAGCTTCG | inverse PCR  inverse PCR | pGD-spGFP1-10-P  pGD-4×spGFP11-P |
| 8 | PGDG R | CATGTGGTCACGCATGGTGGCGACCGGTAGCGCTAGCGCTAG |  |  |
| 9 | spGFP1-10 F | CTACCGGTCGCCACCATGCTCGACCTGCAGATGATCGATAG |  |  |
| 10 | spGFP1-10 R | GATCTGAGTCCGGACTTTTCGTTGGGATCTTTCGAAAGGAC |  |  |
| 11 | PGDG F1 | GATCCCAACGAAAAGTCCGGACTCAGATCTCGAGCTCAAGC | inverse PCR  *Sal*I | pGD-4×spGFP11-P  pGD-4×spGFP11-UBC32 |
| 12 | PGDG R1 | CTGCAGGTCGAGCATGGTGGCGACCGGTAGCGCTAGCGCTAG |  |  |
| 13 | spGFP UBC32 F | GCTCAAGCTTCGAATTCTGCAGTCGACAATACAGCAGCTAGTGTGGTTCC |  |  |
| 14 | spGFP UBC32 R | GGATCCCGGGCCCGCGGTACCGTCGACTCAAGACTGATCATCCATAAACCCAGTACTG |  |  |
| 15 | PET GFP F | GTTTAACTTTAAGAAGGAGATATACATATGATGGGTAAAGGAGAAGAACTTTTC | *Nde*I/*Xho*I | pET-30a-GFP |
| 16 | PET GFP R | GCCGGATCTCAGTGGTGGTGGTGGTGGTGCTTGTATAGTTCATCCATGCCATG |  |  |
| 17 | PET BYS GFP-P F | GTTTAACTTTAAGAAGGAGATATACATATGCACCATCATCATCATCATATGGGTAAAGGAGAAGAACTTTTC | *Nde*I/*Xho*I | pET-30a-GFP-P^WT^, pET-30a-GFP-P^S5A^, pET-30a-GFP-P^S5D^ |
| 18 | PET BYS GFP-P R | GCCGGATCTCAGTGGTGGTGGTGGTGGTGTTAGAGATCTCCATAGGGATCATGAC |  |  |
| 19 | PET32 mCherry F | CATGGACAGCCCAGATCTGGGTACCatggtgagcaagggcgag | *Kpn*I/*Nco*I | pET-32a-mCherry |
| 20 | PET32 mCherry R | gaaaatgagttcatGGATCCGATATCcttgtacagctcgtccatgcc |  |  |
| 21 | mCherry-BY N F | cggcatggacgagctgtacaagGATATCGGATCCATGGCAAAAGAAGATCATGGATTGG | *Bam*HI/*Xho*I | pET-32a-mCherry-N |
| 22 | mCherry-BY N F | GATCTCAGTGGTGGTGGTGGTGGTGGGAGAAGATCTGGTCAGCATTCTTTTTCGCCG |  |  |
| 23 | BYS Trailer F | ATGATAAAAACCATGATTGTTTATAAAACAAATAAATC |  | Cy5-Trailer RNA |
| 24 | T7-BYS Trailer R | TAATACGACTCACTATAGGGACGACCAAGTGAGCCGCAATCTG |  |  |
| 25 | Oligo dT | GGATATCTGCAGGATCCAAGCTTTTTTTTTTTTTTTTTT |  | RT-PCR  (qRT-PCR) |
| 26 | BYS-RT-F | ACGACCAGTGATCGTATAATTTGATTATTGGTGATC |  |  |
| 27 | Trailer-F | GTCTAATAAGCGATGCGTAA |  | Replication  (qRT-PCR) |
| 28 | Trailer-R | CTACCTCTCACACACTCTT |  |  |
| 29 | RFP-F | CCTGGTGGAGTTCAAGTC |  | Transcription  (qRT-PCR) |
| 30 | RFP-R | CACGATGGTGTAGTCCTC |  |  |
| 31 | qNbEF1α-F | AGCTTTACCTCCCAAGTCATC |  | Normal EF1a gene (qRT-PCR) |
| 32 | qNbEF1α-R | AGAACGCCTGTCAATCTTGG |  |  |

Note: F, forward; R, reverse
